# Improved treatment satisfaction and medication adherence after readjusting oral medication regimens with the cooperation of health insurance pharmacy in type 2 diabetic patients in Japan

**DOI:** 10.1186/s40780-017-0096-5

**Published:** 2017-12-06

**Authors:** Naoko Ogawa, Mitsuyoshi Takahara, Toshihiko Shiraiwa, Mayumi Yamamoto, Kaoru Yamamoto, Masayuki Doi, Yoko Yoshida, Setsuko Gotou

**Affiliations:** 1 Shiraiwa Medical Clinic, 4-10-24 Hozenji, Kashiwara City, Osaka 582-0005 Japan; 2 Smile Pharmacy, 4-10-25 Hozenji, Kashiwara City, Osaka 582-0005 Japan

**Keywords:** Treatment satisfaction, Medication adherence, Medication readjustment, Cooperation with health insurance pharmacies

## Abstract

**Background:**

Treatment satisfaction and medication adherence can be improved if physicians carefully monitor the situations, check the level of difficulties patients experience when taking medications at specific times, and readjust medication regimens based on this information. However, physicians in Japan encounter difficulties in taking enough time to collect this information in clinical practice. The aim of the current study was to investigate improvements in satisfaction and adherence with the cooperation of a health insurance pharmacy in clinical practice.

**Methods:**

We retrospectively analyzed 29 type 2 diabetic outpatients who were receiving their prescriptions at a medical clinic and filling prescriptions at a nearby pharmacy. The pharmacy collected information regarding satisfaction, adherence, and preferred time of taking medications, and provided these data to the clinic. The oral medication regimens for these 29 patients were readjusted based on the information obtained.

**Results:**

After readjustments, the dosing frequency was decreased from 3.4 ± 1.2 to 1.8 ± 0.5 times/day, and the number of pills was reduced from 5.7 ± 2.0 to 4.5 ± 1.7 (both *p* < 0.001). Increases in treatment satisfaction from 33 ± 12 to 44 ± 10 points (*n* = 29, *p* < 0.001) were observed when assessed using a questionnaire (60-point maximum). Medication adherence based on pill counts increased from 75% ± 22% to 91% ± 14% (*n* = 24, *p* < 0.001) (5 patients were excluded due to missing data).

**Conclusions:**

Treatment satisfaction and medication adherence were improved after readjustments of oral medication regimens with the cooperation of a health insurance pharmacy in clinical practice in Japan.

## Background

Type 2 diabetes mellitus presents with chronic hyperglycemia due to impaired insulin secretion, as well as insulin resistance [1]. Long-term exposure to hyperglycemia causes a variety of comorbidities including microvascular and macrovascular complications [2], whereas glycemic control can reduce the risk of these complications [3, 4]. Most type 2 diabetic patients are treated with oral antidiabetic drugs (OADs) for glycemic control [5], and adherence to medications is a key factor in the management of type 2 diabetes [6]. However, evidence suggests that adherence to medications in type 2 diabetic patients is less than optimal [7]. Medication adherence is often affected by patient satisfaction with treatment [8], and the importance of improving and maintaining treatment satisfaction has long been emphasized [9]. In some instances, treatment satisfaction and medication adherence can be easily improved if physicians carefully monitor the situations, check the level of difficulties patients experience when taking medications at specific times, and readjust medication regimens based on this information [10]. However, in Japan, consultation times at clinics are often limited [11] and, for many physicians in Japan, taking enough time to collect this information in clinical practice is difficult. In these situations, health insurance pharmacies are expected to effectively contribute to collecting this information. The aim of the current study was to investigate improvements in satisfaction and adherence in type 2 diabetic outpatients with the cooperation of a health insurance pharmacy in clinical practice in Japan.

## Methods

The current retrospective study included 29 Japanese type 2 diabetic outpatients who were treated with OADs and obtained their prescriptions at Shiraiwa Medical Clinic, Osaka, Japan, and filled these prescriptions at a nearby pharmacy, Smile Pharmacy, Osaka, Japan. At the pharmacy, treatment satisfaction and medication adherence were regularly checked and communicated to the clinic prescribing medications. Medication adherence was assessed based on self-reports and/or objective pill counts, whereas treatment satisfaction was evaluated based on a personal interview and monitored using a questionnaire for oral medication satisfaction. These assessments were communicated to the clinic where medications were prescribed as required. In addition, if patients were found experiencing difficulties or issues with taking medications at a specific time and the patients preferred to take medications at another time, the pharmacists simultaneously provided this information to the clinic. Between April and December 2016, a total of 38 type 2 diabetic patients treated at Shiraiwa Medical Clinic had their oral medication regimens changed on the basis of information obtained by the pharmacy. For six patients, the dose of an oral medication other than OADs was changed at the same time, whereas for three patients who were receiving insulin injections, their insulin regimen was changed. The remaining 29 patients were analyzed in the current study to investigate changes in treatment satisfaction and adherence after the readjustment of oral medication regimens. The current retrospective study using medical records was performed in accordance with the Declaration of Helsinki, and was approved by the ethics committee of Shiraiwa Medical Clinic. In accordance with the Ethical Guidelines for Medical and Health Research Involving Human Subjects in Japan, the study was considered exempt from needing informed consent from patients, on the grounds that this was an observational research study using only existing materials and relevant information regarding the study was open to the public.

### Measures of treatment satisfaction and medication adherence

In the current study, a questionnaire-based assessment of treatment satisfaction and a pill count-based evaluation of medication adherence were employed as outcome measures. Treatment satisfaction was evaluated using a questionnaire for oral medication satisfaction [12]. Details regarding the questionnaire were described previously [12]. Briefly, the questionnaire contains a total of 10 statements related to treatment satisfaction and asks to what extent a respondent agrees with each statement. The response is obtained on a 7-point Likert scale, ranging from 0, which corresponds to “strongly disagree,” to 6, which corresponds to “strongly agree.” The 10 statements consist of 6 positively and 4 negatively worded ones. The positive statements include convenience (item no. 1), encouragement of adherence (no. 2), control over diseases (no. 3), feeling of healthiness (no. 4), satisfaction (no. 9), and hopes with receiving treatment (no. 10). On the other hand, the negative statements include inconvenience (no. 5), fear of forgetting to take medications (no. 6), suspicion about efficacy (no. 7), and unfavorably weakened disease awareness (no. 8). The statements are as follows:

1. The current oral treatment is convenient.
2. The current oral treatment encourages adherence.
3. The current oral treatment controls my diseases well.
4. The current oral treatment makes me feel healthy.
5. The current oral treatment is inconvenient.
6. I am worried about forgetting to take medications with the current oral treatment.
7. I am suspicious about the efficacy of the current oral treatment.
8. The current oral treatment undesirably weakens my disease awareness.
9. I am satisfied with the current oral treatment.
10. I hope to continue receiving the current oral treatment.

The questionnaire was originally developed under the concept that all items would, either positively or negatively, reflect a single underlying factor, namely satisfaction with oral treatment, and the calculation of a total score was expected. Validity was confirmed using a factor analysis with a varimax rotation in our preceding study in which 1071 patients with lifestyle-related chronic diseases participated [12]. Accordingly, the questionnaire yields a total satisfaction score by summing the scores from all 10 items, with the rating of the 4 negatively worded items reversed. The possible range in total score was between 0 (no satisfaction) and 60 (full satisfaction) [12].

Objective assessment of medication adherence was based on pill counts. To perform pill counts, the blister packs of dispensed medications were marked using pens for the purpose of distinguishing the currently dispensed medications from previously dispensed but unused ones that patients kept. Patients were asked to take the newly dispensed medications until the next visit, leaving the old unused ones if any. The number of pills patients took was calculated as the number of dispensed pills minus unused pills, and the medication adherence (percentage) was expressed as a ratio of the number of pills consumed to the number of pills expected to be taken between two visits [13]. Note that pill counts were not performed after the readjustment of medication regimens in 5 patients in the study population, and therefore the change in medication adherence was assessed in the remaining 24 patients.

### Statistical analysis

Data are presented as means ± standard deviation (SD) for continuous variables and as percentages for dichotomous variables. A *p* value less than 0.05 was considered to be significant. Changes in a continuous variable were tested using a paired *t* test, whereas dichotomous variables were tested using McNemar’s test, unless otherwise mentioned. Changes in hemoglobin A1c levels were assessed using the last-observational-carried-forward approach. Changes in hemoglobin A1c levels were assessed in patients not receiving insulin therapy. All statistical analyses were performed using IBM SPSS Statistics Version 22 (SPSS Inc., Chicago, IL).

## Results

The clinical characteristics of the study population are shown in Table 1. Patients were 63 ± 12 years old and 69% were male. The mean number of OADs and hemoglobin A1c levels were 2.2 ± 1.0 and 6.8 ± 0.6%, respectively. Prescriptions required the patients to take medications 3.4 ± 1.2 times per day.

**Table 1:** Patient characteristics

*n*	29
Age (years)	63 ± 12
Male	20 (69%)
Durationof diabetes (years)	9 ± 6
Body mass index (kg/m^2^)	25.3 ± 4.0
Hypertension	15 (52%)
Dyslipidemia	20 (69%)
Hemoglobin A1c (%)	6.8 ± 0.6
Number (i.e., type) of OADs (per day)	2.2 ± 1.0
Number (i.e., type) of other oral medications (per day)	1.5 ± 1.1
Total number (i.e., type) of oral medications (per day)	3.8 ± 1.7
Total number of pills (per day)	5.7 ± 2.0
Frequency of taking oral medications (times per day)	3.4 ± 1.2
Combination of Insulin therapy	9 (31%)

Data are shown as means ± SD for continuous variables and n (%) for discrete variables

Based on information derived from the pharmacy, oral medication regimens were readjusted in the clinic to avoid dosing at times patients experienced difficulties in taking medications. Medications were administered alternatively at a different time when patients felt it was easier (or at least less difficult), or were switched to other medications administered at different times. The physicians sometimes made a decision to discontinue a pill administered at a specific time, on the premise that the goal of glycemic control was achieved even though taking the pill was almost always forgotten. A combination product of OADs was also adopted if appropriate. After the readjustment, oral medication regimens were changed as shown in Table 2. The number (i.e., type) of OADs remained unchanged (*p* = 0.537), whereas the frequency of dosing, the total number of pills, and the pattern of administration schedule significantly decreased (all *p* < 0.001). Drug cost was not significantly changed (*p* = 0.348), whereas dispensing fee was significantly decreased (*p* = 0.001). The number of patients taking medications both before and after meals significantly decreased from almost half to zero (*p* < 0.001). Some patients had more OADs decreased in dose than increased, whereas none of the patients had more OADs increased in dose than decreased. The remaining patients either had one OAD increased in dose and the same number decreased, or none increased or decreased. The use of a combination drug became more prevalent, and the total number of pills decreased.

**Table 2:** Changes to oral medication regimens

	Baseline	After changes	P value
Number (i.e., type) of oral drugs (per day)			
OADs	2.2 ± 1.0	2.2 ± 0.8	0.537
Other drugs	1.5 ± 1.1	1.5 ± 1.1	1.000
Type of OADs used			
Sulphonylurea	8 (28%)	6 (21%)	0.500
Alfa-glucosidase inhibitor	11 (38%)	6 (21%)	0.063
Glinide	3 (10%)	2 (7%)	1.000
Biguanide	23 (79%)	25 (86%)	0.500
Thiazolidinedione	1 (3%)	1 (3%)	1.000
Dipeptidyl peptidase-4 inhibitor	19 (66%)	22 (76%)	0.250
Sodium-glucose co-transporter 2 inhibitor	0 (0%)	1 (3%)	1.000
Frequency of taking medications (times/day)	3.4 ± 1.2	1.8 ± 0.5	< 0.001
Taking medications^a^			
In time with all three meals	20 (69%)	2 (7%)	< 0.001
In time with two meals	9 (31%)	17 (59%)	
In time with one meal	0 (0%)	10 (34%)	
Taking medications			
Both before and after meals	14 (48%)	0 (0%)	< 0.001
Only after meals	14 (48%)	21 (72%)	0.016
Only before meals	1 (3%)	8 (28%)	0.039
Change in dose of OADs^b^			
None increased or decreased	–	7 (24%)	–
None increased and one or more decreased	–	12 (41%)	–
One increased and more decreased	–	1 (3%)	–
One increased and one decreased	–	9 (31%)	–
Others	–	0 (0%)	
Use of anti-diabetic combination drug	1 (3%)	8 (28%)	0.016
Total number of pills (per day)	5.7 ± 2.0	4.5 ± 1.7	< 0.001
Pattern of administration schedule^c^	2.2 ± 0.7	1.7 ± 0.6	< 0.001
Pharmacy cost for a 28-day supply (yen)	11,689 ± 5613	10,885 ± 4511	0.0498
Drug cost (yen)	8247 ± 5128	7917 ± 4284	0.348
Dispensing fee (yen)	3442 ± 805	2968 ± 694	0.001

Data are shown as means ± SD for continuous variables and n (%) for discrete variables

^a^The number of meal times was tested using Wilcoxon’s signed rank test

^b^Increases in dose include initiation of a new OAD whereas decreases in dose include discontinuation of an OAD

^c^If a person took some medications before three meals and other medications after breakfast, this was counted as two patterns of administration schedules

After these changes, treatment satisfaction and medication adherence significantly improved (Table 3). In addition, hemoglobin A1c levels decreased at 3 months. No clear adverse events were observed after the readjustment of oral medication regimens in the current study population.

**Table 3:** Change in patient satisfaction, adherence, and glycemic control

	*n*	Baseline	After changes	P value
Treatment satisfaction score (points)	29	33 ± 12	44 ± 10	< 0.001
Medication adherence (%)	24	75 ± 22	91 ± 14	< 0.001
Hemoglobin A1c (%)	20	6.7 ± 0.6(at baseline)	6.5 ± 0.5(at 3 months)	0.025

Data are shown as means ± SD for continuous variables. Changes in medication adherence were assessed in 24 patients since data were not available for 5 patients. Changes in hemoglobin A1c were assessed in 20 patients who were not treated with insulin injections (9 patients treated with insulin therapy were excluded from the analysis)

## Discussion

The results of the current study show an improvement in treatment satisfaction and medication adherence in type 2 diabetic outpatients taking OADs after readjustments of medication regimens with the cooperation of a health insurance pharmacy in clinical practice. The physicians readjusted the patients’ oral medication regimens on the basis of information obtained from the pharmacy regarding treatment satisfaction, medication adherence, and the time at which patients experienced difficulties with taking medications. The frequency of dosing was reduced accordingly, and the total number of pills decreased, with anti-diabetic combination products more frequently used. Treatment satisfaction and medication adherence were found to significantly improve after readjustments.

A majority of type 2 diabetic patients are treated with oral medications; in addition to OADs, and these patients often take medications for other chronic diseases such as hypertension and dyslipidemia. Some OADs need to be taken more than once per day, and the combined use of various medications can result in an increase in dosing frequency. Consequently, frequent dosing is common in clinical practice [12]. Although previous studies suggest that the readjustment of medication regimens, including decreasing dosing frequency, would improve treatment satisfaction and adherence [12, 14, 15], practical approaches in clinical settings have yet to be established. Whether or not an approach is clinically “practical” would be largely dependent on the healthcare system. Approaches which are practical in domestic settings need to be worked out. In Japan, the involvement of health insurance pharmacies can potentially address this issue. Recently, the Ministry of Health, Labour and Welfare presented the “Vision of Community Pharmacies for Patients,” and promoted the extensive involvement of pharmacies in patient healthcare [16]. The current report would be a successful and therefore important precedent for the practical involvement of pharmacies in clinical settings in Japan.

The involvement of the health insurance pharmacy in the current study included collection of information about patient satisfaction, adherence, and preferred time for taking medications, which was then reported to the clinic. The physicians in the clinic performed readjustments of oral medication regimens based on the information received from the pharmacy.

The approach may be quite simple, and may not be new at all. However, in Japan, consultation times at clinics are often limited [11] and many physicians find it difficult to schedule enough time to collect this information from all patients in real-world clinical settings. The cooperation of pharmacies in collecting this information would serve a breakthrough in clinical practice in Japan.

The current study observed a significant improvement of a questionnaire-assessed treatment satisfaction after the approach. Note that the questionnaire had no absolute threshold, and therefore the assessment was relative, rather than absolute. It remained to be known whether patients would became absolutely satisfied with the readjusted medication regimens. In addition, the current study was conducted in a retrospective, observational manner and therefore we cannot draw an ultimate conclusion regarding which factors would be a true cause of the improvement. However, one possible explanation of the current observation is that treatment satisfaction would be improved by avoiding administration of medications at times when patients experienced difficulties in taking medications. This direct readjustment would make their oral treatment more convenient, leading to the improvement of treatment satisfaction. In addition, the current readjustment of medication regimens included the decrease of dosing frequency, which might also improve treatment satisfaction. Indeed, reducing dosing frequency has often been emphasized [14]. However, patient satisfaction would not change if dosing was reduced in frequency but patients still took medications at times when they encountered difficulties. The decrease of dosing frequency would not be successful in the improvement of treatment satisfaction unless patients’ difficulties in taking medications were relieved. The information about preferred times for taking medication would make a great contribution to the success of this strategy.

The improvement of medication adherence could be interpreted to accompany the improvement of treatment satisfaction. It is well recognized that patients’ satisfaction with treatment is a major determinant of adherence [8]. The medication regimens became more convenient for patients after the readjustment, and that would promote medication adherence. Administration of medications at the time preferred by patients would directly improve the medication adherence. At the same time, we must note that the medication adherence was assessed by pill counts in the current study population. It was possible that the repletion of the survey itself would become an intervention and affect medication adherence [17], although the influence was so far controversial [18]. Future prospective studies with a control arm will be needed to conclude that the readjustment would be a cause of the improvement of medication adherence. Despite this study limitation, however, we believe that it was clinically important that both medication adherence and treatment satisfaction were simultaneously improved in the current study population. Previous studies suggest that medication adherence may be improved by interventions without readjusting dosing regimens, such as counselling patients about the importance of adherence and reminding patients to take medications through phone calls and/or e-mail communications [19]. However, it remains unclear whether those approaches would also lead to improvements in treatment satisfaction. It is true that, in one aspect, treatment satisfaction is just a factor that affects medication adherence. However, in another aspect, satisfaction directly affects patient quality of life (QOL), the ultimate goal in anti-diabetic treatment. Life-long QOL would not be gained but be lost if patients achieve significant glycemic control but feel burdened with their treatment [20]. It would be important to readjust medication regimens to improve satisfaction, even if hyperglycemia is well-controlled. The improvement of treatment satisfaction and adherence would be also due partly to the decrease in total pill number, promoted by the use of combination products [21]. Since combination drugs were introduced simultaneously with the readjustment of dosing time in this retrospective study population, this factor’s independent contribution to patient satisfaction and adherence remained unknown. Future clinical trials are needed to strictly distinguish between the improvement due to time readjustment and that due to the use of combination products.

In the current study, we also observed a significant reduction of hemoglobin A1c levels three months after readjustment, even though a substantial number of the population had their anti-diabetic medications decreased rather than increased in dose, and none of the patients had their medications increased rather than decreased. The current study did not have a control group set, and the possibility that the changes in hemoglobin A1c levels observed in the current study may have been influenced by various confounding factors cannot be ruled out. Nonetheless, it is noteworthy that glycemic control did not, at least, deteriorate after readjustments. One possible explanation is that the improved medication adherence would enable OADs to bring a sufficient pharmacological (i.e., glucose-lowering) effects, leading to better glycemic control in the current study population [22]. The current study assessed treatment satisfaction and medication adherence for both OADs and other medications, whereas the efficacy of treatment was only evaluated using hemoglobin A1c levels in a diabetes-specific manner. Future studies will be needed for a comprehensive understanding of the association among treatment satisfaction, medication adherence, and treatment efficacy.

The current study had some limitations. First, the current study was a retrospective, single-center, non-controlled study. Second, the sample size was small. Third, although the pharmacists confirmed that the current study patients had no records of filling medications at other pharmacies during the study period, the pharmacists may have failed to recognize the medication filled without any record in other pharmacies. In addition, data were unavailable on the number of pills patients received before pill counts that were unused and left at home. Further studies will be needed to validate the current findings.

## Conclusions

The results of the current study show improvements in treatment satisfaction and medication adherence in type 2 diabetic outpatients taking OADs after readjustments of medication ragimens with the cooperation of a health insurance pharmacy in clinical practice. Treatment satisfaction and medication adherence significantly improved after the physicians readjusted the patients’ oral medication regimens on the basis of information obtained from the pharmacy regarding treatment satisfaction, medication adherence, and the time at which patients experienced difficulties in taking medications.

## Abbreviations

OAD: Oral antidiabetic drug
QOL: Quality of life
SD: Standard deviation

## References

[1] Seino Y, Nanjo K, Tajima N, Kadowaki T, Kashiwagi A, Araki E (2010). Report of the committee on the classification and diagnostic criteria of diabetes mellitus. J Diabetes Investig.

[2] Stratton IM, Adler AI, Neil HA, Matthews DR, Manley SE, Cull CA (2000). Association of glycaemia with macrovascular and microvascular complications of type 2 diabetes (UKPDS 35): prospective observational study. BMJ.

[3] UK Prospective Diabetes Study (UKPDS) Group (1998). Intensive blood-glucose control with sulphonylureas or insulin compared with conventional treatment and risk of complications in patients with type 2 diabetes (UKPDS 33). Lancet.

[4] Holman RR, Paul SK, Bethel MA, Matthews DR, Neil HA (2008). 10-year follow-up of intensive glucose control in type 2 diabetes. N Engl J Med.

[5] Yokoyama H, Oishi M, Takamura H, Yamasaki K, Shirabe SI, Uchida D (2016). Large-scale survey of rates of achieving targets for blood glucose, blood pressure, and lipids and prevalence of complications in type 2 diabetes (JDDM 40). BMJ Open Diabetes Res Care.

[6] Ho PM, Rumsfeld JS, Masoudi FA, McClure DL, Plomondon ME, Steiner JF (2006). Effect of medication nonadherence on hospitalization and mortality among patients with diabetes mellitus. Arch Intern Med.

[7] Krass I, Schieback P, Dhippayom T (2015). Adherence to diabetes medication: a systematic review. Diabet Med.

[8] Barbosa CD, Balp MM, Kulich K, Germain N, Rofail D (2012). A literature review to explore the link between treatment satisfaction and adherence, compliance, and persistence. Patient Prefer Adherence.

[9] Reaney M, Elash CA, Litcher-Kelly L (2016). Patient reported outcomes (PROs) used in recent phase 3 trials for type 2 diabetes: a review of concepts assessed by these PROs and factors to consider when choosing a PRO for future trials. Diabetes Res Clin Pract.

[10] Rubin RR (2005). Adherence to pharmacologic therapy in patients with type 2 diabetes mellitus. Am J Med.

[11] Kabeya Y, Uchida J, Toyoda M, Katsuki T, Oikawa Y, Kato K (2017). Factors affecting consultation length in a Japanese diabetes practice. Diabetes Res Clin Pract.

[12] Takahara M, Shiraiwa T, Ogawa N, Yamamoto M, Kusuda Y, Shindo M (2017). Patient perspectives on combination therapy of a once-weekly oral medication plus daily medication for lifestyle-related chronic diseases. Intern Med.

[13] Lam WY, Fresco P (2015). Medication adherence measures: an overview. Biomed Res Int.

[14] Srivastava K, Arora A, Kataria A, Cappelleri JC, Sadosky A, Peterson AM (2013). Impact of reducing dosing frequency on adherence to oral therapies: a literature review and meta-analysis. Patient Prefer Adherence.

[15] Falagas ME, Karagiannis AK, Nakouti T, Tansarli GS (2015). Compliance with once-daily versus twice or thrice-daily administration of antibiotic regimens: a meta-analysis of randomized controlled trials. PLoS One.

[16] Ministry of Health Law. Vision of Community Pharmacies for Patients. http://www.mhlw.go.jp/file/04-Houdouhappyou-11121000-Iyakushokuhinkyoku-Soumuka/vision_1.pdf. Accessed 8 May 2017.

[17] Achieng L, Musangi H, Billingsley K, Onguit S, Ombegoh E, Bryant L (2013). The use of pill counts as a facilitator of adherence with antiretroviral therapy in resource limited settings. PLoS One.

[18] Lindenmeyer A, Hearnshaw H, Vermeire E, Van Royen P, Wens J, Biot Y (2006). Interventions to improve adherence to medication in people with type 2 diabetes mellitus: a review of the literature on the role of pharmacists. J Clin Pharm Ther.

[19] Nieuwlaat R, Wilczynski N, Navarro T, Hobson N, Jeffery R, Keepanasseril A, et al. Interventions for enhancing medication adherence. Cochrane Database Syst Rev. 2014: CD000011. doi:10.1002/14651858.CD000011.pub4. 10.1002/14651858.CD000011.pub4 PMC7263418 25412402

[20] Vijan S, Sussman JB, Yudkin JS, Hayward RA (2014). Effect of patients’ risks and preferences on health gains with plasma glucose level lowering in type 2 diabetes mellitus. JAMA Intern Med.

[21] Hutchins V, Zhang B, Fleurence RL, Krishnarajah G, Graham J (2011). A systematic review of adherence, treatment satisfaction and costs, in fixed-dose combination regimens in type 2 diabetes. Curr Med Res Opin.

[22] Kardas P (2005). The DIACOM study (effect of DosIng frequency of oral Antidiabetic agents on the COMpliance and biochemical control of type 2 diabetes). Diabetes Obes Metab.

